# Triblock copolymer micelles enhance solubility, permeability and activity of a quorum sensing inhibitor against *Pseudomonas aeruginosa* biofilms†

**DOI:** 10.1039/d3lp00208j

**Published:** 2024-02-27

**Authors:** Karolina Kasza, Fadi Soukarieh, Manuel Romero, Kim R. Hardie, Pratik Gurnani, Miguel Cámara, Cameron Alexander

**Affiliations:** a Division of Molecular Therapeutics and Formulation, School of Pharmacy, University of Nottingham NG7 2RD UK cameron.alexander@nottingham.ac.uk; b National Biofilms Innovation Centre, School of Life Sciences, Biodiscovery Institute, University Park, University of Nottingham Nottingham NG7 2RD UK; c Department of Microbiology and Parasitology, Faculty of Biology-CIBUS, Universidade de Santiago de Compostela Santiago de Compostela 15782 Spain; d UCL School of Pharmacy, University College London 29-39 Brunswick Square London WC1N 1AX UK; e Aquatic One Health Research Center (ARCUS), Universidade de Santiago de Compostela Santiago de Compostela, 15782 Spain

## Abstract

Antimicrobial resistance is a threat to public health for which new treatments are urgently required. The capability of bacteria to form biofilms is of particular concern as it enables high bacterial tolerance to conventional therapies by reducing drug diffusion through the dense, exopolymeric biofilm matrix and the upregulation of antimicrobial resistance machinery. Quorum sensing (QS), a process where bacteria use diffusible chemical signals to coordinate group behaviour, has been shown to be closely interconnected with biofilm formation and bacterial virulence in many top priority pathogens including *Pseudomonas aeruginosa*. Inhibition of QS pathways therefore pose an attractive target for new therapeutics. We have recently reported a new series of *pqs* quorum sensing inhibitors (QSIs) that serve as potentiators for antibiotics in *P. aeruginosa* infections. The impact on biofilms of some reported QSIs was however hindered by their poor penetration through the bacterial biofilm, limiting the potential for clinical translation. In this study we developed a series of poly(β-amino ester) (PBAE) triblock copolymers and evaluated their ability to form micelles, encapsulate a QSI and enhance subsequent delivery to *P. aeruginosa* biofilms. We observed that the QSI could be released from polymer micelles, perturbing the *pqs* pathway in planktonic *P. aeruginosa*. In addition, one of the prepared polymer variants increased the QSIs efficacy, leading to an enhanced potentiation of ciprofloxacin (CIP) action and therefore improved reduction in biofilm viability, compared to the non-encapsulated QSI. Thus, we demonstrate QSI encapsulation in polymeric particles can enhance its efficacy through improved biofilm penetration.

## Abstract 2

Combination therapies offer potential solutions to the growing threats of antimicrobial resistance. We describe polymer delivery materials for Quorum Sensing Inhibitors used in combination with ciprofloxacin to enhance potency in bacterial biofilms.

## Graphical abstract

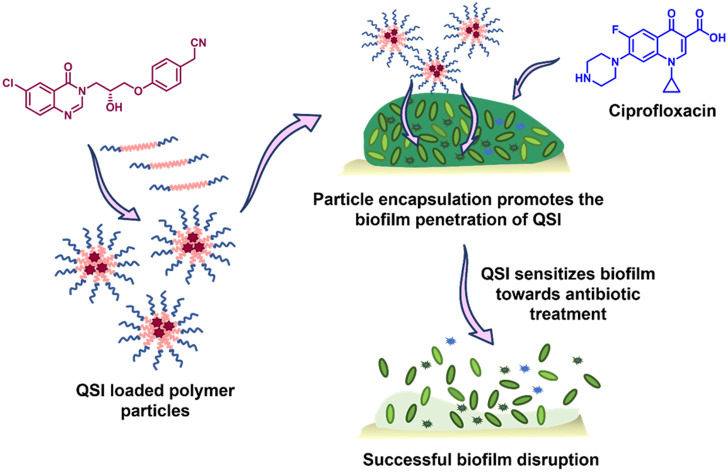

## Introduction

Bacterial biofilms, composed of cell clusters suspended in a thick extracellular matrix, pose a substantial healthcare challenge, due to their high resilience to conventional therapies, caused by limited therapeutic penetration through the biofilm matrix and upregulation of resistance pathways.^1–3^ To address this issue, novel antimicrobial approaches are urgently required, with the targeting of quorum sensing (QS) pathways being of particular interest, due to their role in antimicrobial tolerance and biofilm maturation. QS has been reported to govern the expression of diverse genes responsible for antimicrobial tolerance and ‘community control’ through the excretion of small chemical signals known as autoinducers.^4,5^ QS use has been particularly explored in *Pseudomonas aeruginosa*, a World Health Organisation priority pathogen, linked to prevalent infections, such as those present in the cystic fibrosis lung, chronic wounds and chronic obstructive pulmonary diseases.^6–8^ Biofilm formation within *P. aeruginosa* is a tightly controlled phenotype by a complex regulatory network, incorporating three types of QS systems employing various types of autoinducer signal molecules.^9^ Within those the *las* and *rhl* systems use *N*-acylhomoserine lactone derived signals, while the *pqs* system relies on alkyl quinolone derived molecules.^10^ The *pqs* system has been shown to play a significant role in the virulence and biofilm maturation of *P. aeruginosa* therefore making it an attractive target for novel antimicrobial therapies.^11^ The biosynthesis of the Pseudomonas Quinolone Signal (PQS) in *P. aeruginosa* is controlled by the *pqsABCDE* operon, positively regulated by the LysR-type transcriptional regulator PqsR upon binding PQS and repressed by the transcriptional regulator protein RhlR.^12^ Successful inhibition of QS can therefore be achieved through the pharmacological inhibition of the PqsR–PQS interaction, hence leading to a decrease in the operon expression and quinolone signal production. To address this, we recently reported a high throughput chemical and *in silico* screening of inhibitors for the *pqs* system, resulting in the discovery of several potent quorum sensing inhibitors (QSIs) that could alleviate recalcitrant infections caused by *P. aeruginosa* when administered in combination with the antibiotic ciprofloxacin (CIP).^10,13,14^ Particularly high potency was observed for (*R*)-2-(4-(3-(6-chloro-4-oxoquinazolin-3(4*H*)-yl)-2-hydroxypropoxy)phenyl)acetonitrile (QSI61), (hit compound 61 within the screen^10^) however its *in vitro* anti-biofilm activity was hindered by the poor penetration of the molecule through the thick exopolymeric matrix.

Drug delivery can be applied to overcome many of the challenges associated with small molecule therapies, either by enhancing therapeutic efficacy or improving patient tolerability by minimising adverse side effects, with the use of polymer-based systems favoured by their versatility and ease of modification.^15,16^ To date a plethora of polymer scaffolds has been reported for the delivery of antibiotics to biofilms, including biodegradable materials such as poly(lactic-*co*-glycolic acid) (PLGA)^17^ and poly(caprolactone)^18^ and non-degradable polymers including poly(ethylene glycol),^19^ and poly(methacrylates).^20^ Reports on the polymer delivery of QSIs have been more limited. Kang *et al.* reported the sustained delivery of Furanone C-30 in PLGA microparticles, with successful inhibition of *Streptococcus mutans* growth demonstrated.^21^ Singh *et al.* demonstrated an improved efficacy of 3-amino-7-chloro-2-nonylquinazolin-4(3*H*)-one conjugated to alginate nanoparticles loaded with CIP, with pH responsive release of the antimicrobials demonstrated once in the acidic environment of the biofilm.^22^ More recently, we showed an improved biofilm penetration of QSI61 when conjugated by esterase cleavable linkers to non-biodegradable amphiphilic polyacrylates, with a substantial improvement upon non-conjugated QSI activity observed in *P. aeruginosa* biofilms 6 h following treatment.^23^ Considering the promising efficacy reported for the RAFT–QSI conjugates it is therefore of interest to explore further methods for QSI61 delivery, aside from its conjugation to non-biodegradable linear polymers. Drug encapsulation within a hydrophobic micelle core is of particular interest due to its release mechanism being independent of esterase action. Moreover, the use of biodegradable polymers would further lower the risk of systemic toxicity from the delivery system.

We therefore hypothesised that the anti-biofilm efficacy of our QSI61 could be enhanced through its encapsulation within biodegradable polymeric particles, leading to an improvement in biofilm penetration and hence potentiating the CIP treatment against *P. aeruginosa* communities. Within the field of biodegradable polymers, poly(β-amino esters) (PBAEs), are of particular interest due to their pH-responsive behaviour, controlled through the protonation of the polymers’ central nitrogen atoms and facile synthesis through a one-pot aza-Michael addition of primary or secondary amines to diacrylates.^24^ Despite these advantages, the use of PBAEs as hydrophobic cores in polymeric micelles has to date been limited, due to the sparse approaches towards PBAE end group modification. To address this our group recently reported a *grafting-from* methodology for PBAE post-synthetic functionalisation with RAFT polymers, enabling the synthesis of a diverse range of ABA triblock copolymer scaffolds.^25^

**Fig. 1:**
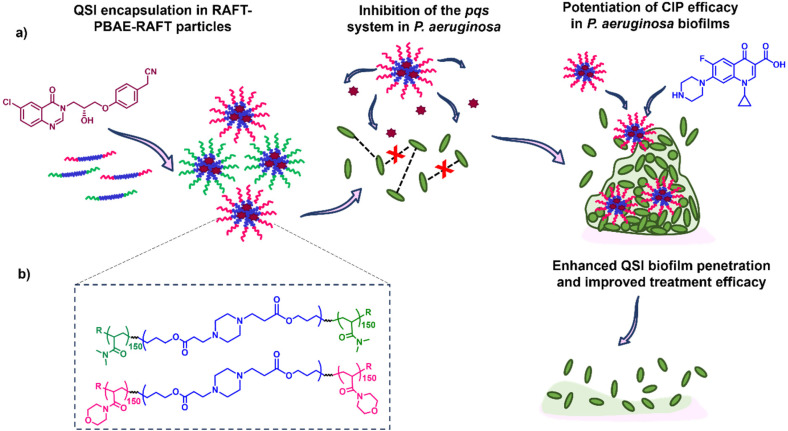
Experimental design overview where the hydrophobic QSI61 is encapsulated in RAFT-PBAE-RAFT polymeric particles and subsequently released to inhibit quorum sensing pathways in planktonic *P. aeruginosa* while promoting the antimicrobials biofilm penetration and hence efficacy in mature *P. aeruginosa* biofilms; where: (a) outline of the study including structures of the QSI (purple) and antibiotic (blue) used; (b) structure of the RAFT-PBAE-RAFT particles used for the testing.

Here, we report the application of our RAFT-PBAE-RAFT micelle platform for the delivery of QSI61 to *P. aeruginosa* biofilms with the aim of improving its biofilm penetration. To achieve this, we synthesised PBAEs based on hexanediol diacrylate (HDD) and piperazine (PIP) followed by copolymerization with two types of RAFT monomers: *N*-acryloyl morpholine (NAM) and *N*,*N*-dimethylacrylamide (DMA). The triblock copolymers were then formulated into nanoparticles with sizes below 200 nm and loaded with the QSI. The suitability of the particle carriers was evaluated in a series of assays including the quantification of QSI release from polymer micelles, testing of the effect on QS in *P. aeruginosa*, evaluation of the particle's biofilm penetration and the reduction in mature biofilm viability when administered in combination with free CIP. We observed our NAM_150_-(HDD-PIP)-NAM_150_ particles improved upon free QSI efficacy, leading to an enhanced potentiation of CIP activity and therefore increased reduction in biofilm viability. Hence, we demonstrate QSI61 encapsulation within RAFT-PBAE-RAFT polymeric particles is a promising strategy to promote QSI activity by improving upon its biofilm penetration (Fig. 1).

## Materials and methods

### Materials

Hexanediol diacrylate (HDD), piperazine (PIP), triethylamine (TEA), dimethyl sulfoxide (DMSO)-d6 (99.5% D atom), chloroform-d (99.8% D atom), 4,4′-azobis(4-cyanovaleric acid) (ACVA, >98%), and esterase from porcine liver were obtained from Sigma-Aldrich without further purification. *N*-Acryloyl morpholine (NAM) and *N*,*N*-dimethylacrylamide (DMA), were purchased from Sigma-Aldrich and the inhibitor removed by passing the monomers through a column of basic aluminium oxide. Acryloxyethyl thiocarbamoyl Rhodamine B was purchased from Polysciences, Inc. Solvents and other reagents were acquired from commercial sources and used as received unless stated otherwise.

2-(((Butylthio)carbonothioyl)thio)propanoic acid (PABTC), *N*-hydroxysuccinamide-(propanoic acid)yl butyl trithiocarbonate (NHS-PABTC) and (*R*)-2-(4-(3-(6-chloro-4-oxoquinazolin-3(4*H*)-yl)-2-hydroxypropoxy)phenyl)acetonitrile (QSI61) were synthesised by methods previously reported in literature.^10,10,26,27^

#### Bacterial strains

*Pseudomonas aeruginosa* (Gram-negative, PAO1-L, Lausanne collection wild type *P. aeruginosa* strain, Holloway collection, *via* D. Haas^28^), was used for the microbial assays.

### Methods

#### Instrumentation and analysis

##### NMR spectroscopy

^1^H NMR spectra were recorded on a Bruker DPX-400 spectrometer using dimethyl sulfoxide (DMSO)-d_6_ (99.5% D atom) or chloroform-d (99.8% D atom).

##### Size exclusion chromatography (SEC)

A Polymer Laboratories PL-50 instrument equipped with differential refractive index (DRI) was used for SEC analysis. The system was fitted with 2 × PLgel mixed D columns (300 × 7.5 mm) and a PLgel 5 μm guard column. The eluent used was DMF with 0.1% LiBr. Samples were run at 1 mL min^−1^ at 50 °C. Poly(methyl methacrylate) standards (Agilent EasyVials) were used for calibration between 955 500–550 g mol^−1^. Analyte samples were filtered through a membrane with 0.22 μm pore size before injection. Experimental molar mass (*M*_n_, SEC) and dispersity (*Đ*) values of synthesised polymers were determined by conventional calibration using Cirrus GPC software.

##### Dynamic light scattering

Dynamic light scattering (DLS) measurements were measured using a Malvern Zetasizer Nano ZS apparatus equipped with a He–Ne laser operated at *λ* = 633 nm and at a scattering angle of 173°. Particle size was measured at concentrations of 1 mg mL^−1^ in water at 25 °C, with three scans taken per measurement.

### Theoretical molar mass calculation

Calculation of theoretical number average molar mass (*M*_n,th_) where [M]_0_ and [CTA]_0_ are the initial concentrations (in mol dm^−3^) of monomer and chain transfer agent respectively. *p* is the monomer conversion as determined by ^1^H NMR spectroscopy. *M*_M_ and *M*_CTA_ are the molar masses (g mol^−1^) of the monomer and chain transfer agent respectively. Used to calculate the theoretical molar mass of the PBAE-RAFT ABA triblock copolymers.1
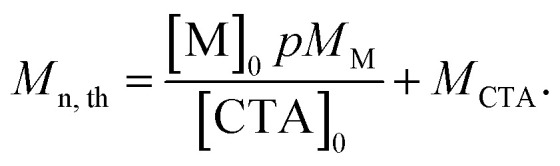


### PBAE synthesis

PBAEs were synthesised as previously reported.^29^ Briefly, hexanediol diacrylate (10 g, 44.2 mmol) was mixed with piperazine at a 1.1 : 1 molar ratio of monomer to amine in dioxane at 500 mg mL^−1^ and the reaction stirred in the dark at 90 °C for 24 h. Following reaction completion, the mixture was diluted (167 mg mL^−1^) and end-capped using 2,2-(ethylenedioxy)diethylamine (0.5 M) at 25 °C for 24 h. The resulting polymer was purified in tetrahydrofuran (THF), and diethyl ether (1 : 9) and the solvent removed under reduced pressure to yield HDD-PIP as a yellow, viscous liquid. Amine capping efficacy was assessed using ^1^H NMR with no acrylate peaks present following the capping step. The final polymers were characterised by SEC and ^1^H NMR.

### PBAE functionalisation with NHS-PABTC

PBAE functionalisation with NHS-PABTC was conducted as previously reported in literature.^25^ Briefly HDD-PIP (1 g, 1 eq.) and NHS-PABTC (6 eq.) were solubilized in DMF (1 mg mL^−1^ final PBAE concentration) following which TEA (3 eq.) was added. The reaction was left to stir (450 rpm, 25 mm stirrer bar) in the dark at 25 °C for 48 h. Following reaction completion, the resulting PBAE–*m*CTA was purified in THF and diethyl ether (1 : 9), and the solvent removed under reduced pressure yielding the HDD-PIP-*m*CTA as a yellow, viscous liquid. The final polymer was analysed by SEC and ^1^H NMR.

### Grafting-from RAFT polymerisation

Grafting-from RAFT polymerisation was conducted as previously described.^25^ RAFT polymer chain extension was conducted in dioxane under nitrogen with the NAM and DMA monomers (1.5 M), selecting a degree of polymerisation (DP) of 300; using ACVA (10 mg mL^−1^ stock in dioxane) as the initiator and keeping the CTA to initiator ratio as 2. The reaction was left to stir under nitrogen at 70 °C for 24 h. The resulting polymers (NAM_150_-(HDD-PIP)-NAM_150_ and DMA_150_-(HDD-PIP)-DMA_150_) were analysed using ^1^H NMR and SEC. Conversion was assessed using ^1^H NMR by comparing the integration of the acrylate/acrylamide peaks before and after reaction completion, using the PBAE polyester protons a 4.01 ppm as a reference. Rhodamine tagged polymers were synthesised by adding a stock solution of acryloxyethyl thiocarbamoyl rhodamine B in DMF (10 μg mL^−1^) to the reaction mixture, targeting a 0.1% molar dye content in total number of monomer moles used.

### PBAE-RAFT particle formulation by direct solubilization in water

Micelles were formulated by adding Mili-Q grade water (5 mL) to dry polymer (5 mg) in a scintillation vial (20 mL), under constant stirring (960 rpm, 25 mm stirrer bar) and suspension being left to stir for 1 h, to yield a final polymer concentration of 1 mg mL^−1^.

### QSI encapsulation

Mili-Q grade water (5 mL) and a QSI solution in dimethyl sulfoxide (DMSO) (0.5 mL, 1 mg mL^−1^) were simultaneously added to weighed out polymer (5 mg) and left to stir (960 rpm, 25 mm stirrer bar) for 2 h to yield a final polymer concentration of 1 mg mL^−1^. Centrifugal filtration (3500 Dalton molecular weight cut-off, Amicon) was applied to purify the unencapsulated drug. The experiments were repeated three times, each time using three technical replicates.

### Quantification of drug load by HPLC

QSI loaded particles (5 mL) were freeze dried for 24 h (0.98 millibar), dissolved in a 50 : 50 mixture of DMSO and trifluoroacetic acid (TFA) and left to stir for 3 h. The solution was then diluted 1 : 10 in DMSO and QSI encapsulation levels assessed by High Performance Liquid Chromatography (HPLC) (Agilent technologies 1200 series, USA). The experiment was repeated three times, each time using three technical replicates.

QSI loading was assessed using a C18 (4.6 × 250 mm; 5 μm) analytical column (ZORBAX Eclipse Plus). The UV detector was operated at 275 nm. The eluting gradient consisted of a pre-equilibration run at 10% B, increasing to 90% B following 10 min, then 10% B following 20 min and remaining at 10% B for a further 5 min (where solvent A is 0.1% formic acid in water and solvent B is 0.1% formic acid in acetonitrile). The flow rate was set at 1.0 mL min^−1^ and injection volume at 20 μL.^10^

Drug loading and encapsulation efficiency were calculated using the following equations:

Calculation of percentage drug load.2
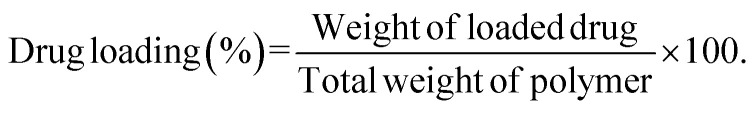


Calculation of percentage drug encapsulation.3
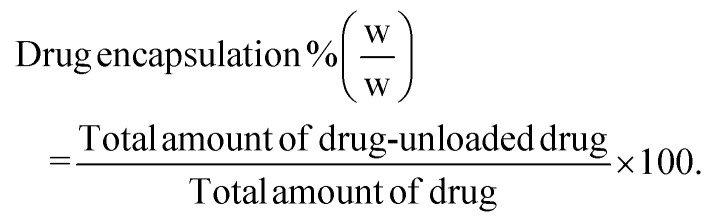


### QSI release study

QSI release from polymer carrier was assessed in phosphate buffer at pH 7.4, containing 0.01% Tween 20. A sample (1 mL, 6.6 mg mL^−1^) of QSI-loaded nanoparticles was placed in a dialysis membrane (3500 Dalton molecular weight cut-off, Spectrum Labs) The micellar solution was dialysed against 30 mL of release media at 37 °C, over 48 h, and samples (1 mL) were collected at appropriate timepoints and replaced with the same amount of fresh medium to maintain sink conditions. QSI quantification from the release samples was conducted by HPLC as described above. For esterase triggered release testing esterase from porcine liver (1 mg mL^−1^) was added to the release sample.

### *Pqs* biosensor reporter assay

The activity of the particle-loaded QSI in planktonic bacteria was evaluated using the bioreporter strain *P. aeruginosa* PAO1-L *mCTX*::P_*pqsA*_-*lux* as previously described.^14^ For testing, the particle-loaded and free QSI were assessed at concentrations of 50, 5 and 0.5 μM, prepared from 50 mM DMSO stocks for free QSI and compared to an equivalent DMSO control.

### Checkerboard combined antimicrobial efficacy assay

The CIP-QSI and polymer-QSI synergistic effect was assessed in a checkerboard assay in a Nunclon 96-well plate using a 5-by-4 well configuration. Briefly single *P. aeruginosa* colonies were used to inoculate 5 mL Lysogeny Broth (LB) in sterile universal tubes. Overnight cultures were grown at 37 °C in a shaking (200 rpm) incubator. After overnight growth, each culture was diluted to an OD_600 _value of 0.02 using LB. 100 μL of the diluted culture was treated with 100 μL treatment solution diluted in LB. QSI concentrations between 10 and 1.25 μM were tested and combined with CIP concentrations ranging from 0.25 to 0.016 μg mL^−1^ or NAM_150_-(HDD-PIP)-NAM_150_ or DMA_150_-(HDD-PIP)-DMA_150_ polymers at concentrations between 1 and 0.06 mg mL^−1^. Following treatment, the bacteria were incubated at 37 °C for 24 hours, with OD_600 _measurements for each well recorded at *T*0 and *T*24 h. OD_600 _measurements of the *T*24 h timepoint were then used to calculate the percentage of bacterial inhibition using eqn (4) and the obtained value subtracted from 100 to obtain the percentage survival. The obtained concentration curves were then compared to observe potential synergistic action. Each experiment was repeated in triplicate.

Calculation of % inhibition, where *A*_CP_ is the absorbance of the positive control (no polymer), *A*_CN_ is the absorbance of the negative control (LB only), and *A*_S_ is the absorbance of the tested sample.^30^

4 % inhibition = [1 − (*A*_s_ − *A*_CN_)/(*A*_CP_ − *A*_CN_)] × 100

### Biofilm viability and polymer penetration studies

RAFT-PBAE-RAFT particle diffusion through the biofilm matrix was assessed by exposing mature 1-day old *P. aeruginosa* PAO1-L biofilms to Rhodamine labelled particles administered at concentrations of 1 mg mL^−1^. Following 4 h the biofilm samples were collected and imaged using a LSM700 AxioObserver (Carl Zeiss, Germany) confocal laser scanning microscope (CLSM). Biofilm samples were collected and stained with Syto9 fluorescent dye prior to CLSM image acquisition to simultaneously detect bacterial cells (green spectra range) and polymeric particles (red spectra range). RAFT-PBAE-RAFT particle diffusion was assessed by image analysis of the resulting biofilms with saturation of lower biofilm levels detected.

Effect of particle encapsulation on QSI activity was evaluated in mature 1-day old *P. aeruginosa* PAO1-L biofilms. Biofilms were grown as previously described^31^ on round glass coverslips (13 mm diameter, #1.5 mm thickness) under dynamic conditions (20 rpm) in FAB 10 mM glucose medium^32^ and inoculated with diluted (OD_600 nm_ = 0.01) *P. aeruginosa* (PAO1-L) from overnight cultures in LB. Following 24 h cultivation at 30 °C the biofilms were washed in phosphate buffered saline (PBS) to remove loosely attached cells and incubated for further 24 h in fresh medium supplemented with various treatments. These included free CIP (60 μg mL^−1^), QSI (10 μM) loaded NAM_150_-(HDD-PIP)-NAM_150_ and DMA_150_-(HDD-PIP)-DMA_150_ particles with and without CIP addition (60 μg mL^−1^), non-encapsulated QSI with and without CIP (60 μg mL^−1^) and NAM_150_-(HDD-PIP)-NAM_150_ and DMA_150_-(HDD-PIP)-DMA_150_ particles with no drug loaded. Biofilms exposed to each treatment were washed in PBS and the viability of attached cells evaluated by fluorescent staining using the LIVE/DEAD® BacLight™ Bacterial Viability kit (Molecular Probes, Life Technologies) according to manufacturer instructions. Following staining, coverslips were rinsed with distilled water and imaged using CLSM. The quantification of viable and non-viable biofilm mass was done using Fiji-ImageJ software from the imaged biofilm stacks. Live/dead ratios were established for each treatment and compared to untreated controls to obtain percentage changes in biofilm viability.

## Results and discussion

### Polymer synthesis and characterization

**Fig. 2:**
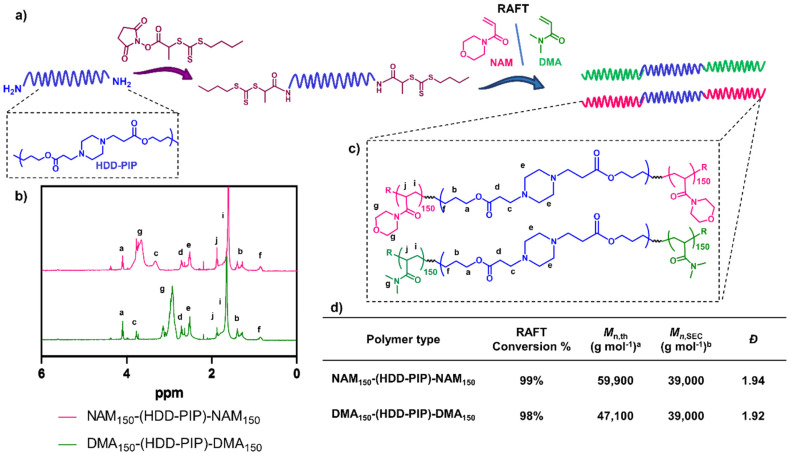
Synthesis outline and polymer characterization. (a) Outline of the synthetic methodology to synthesise RAFT-PBAE-RAFT triblock copolymers; (b) ^1^H NMR spectra in CDCl_3_ of NAM_150_-(HDD-PIP)-NAM_150_ and DMA_150_-(HDD-PIP)-DMA_150_ polymers; (c) structures of both polymers; (d) inset table with polymer ^1^H NMR and SEC characterisation ^*a*^ calculated using eqn (2). ^*b*^ Determined using DMF-SEC.

The RAFT-PBAE-RAFT polymers were prepared using previously reported methods.^29^ Briefly the PBAE was synthesised from hexanediol diacrylate (HDD) and piperazine (PIP) *via* traditional one-pot aza-Michael addition chemistry employing a 1.1 : 1 diacrylate : amine ratio.^29^ The resulting polymer showed residual acrylate signals in the ^1^H NMR spectra and a monomodal mass distribution. The terminal acrylates were then end-capped with an excess of (2,2-(ethylenedioxy)diethylamine) to avoid any PBAE coupling, with full amine functionalisation confirmed by the complete disappearance of the acrylate signals in the ^1^H NMR spectra. An increase in molar mass to *M*_n,SEC_ = 14 000 g mol^−1^ was observed following polymer purification in a mixture of THF and cold ether (1 : 9), hypothesised to be caused by the removal of lower mass molar chains due to their poor solubility in the purification solvent. Polymer functionalisation into a macromolecular chain transfer agent (HDD-PIP-mCTA) was then undertaken at the α and ω end groups with an *N*-hydroxysuccinimide functional RAFT agent, NHS-PABTC (Fig. 2a). This approach to PBAE functionalisation with NHS-PABTC allowed for the subsequent attachment of RAFT copolymer at both the α and ω end groups, thus leading to the synthesis of ABA triblock copolymers. Following CTA functionalisation the *M*_n,SEC_, was shown to increase to 18 000 g mol^−1^, which we again attribute to the solubilisation of lower molar mass chains in the purification solvent. RAFT *grafting-from* polymerization was then performed to obtain triblock ABA copolymers, targeting a degree of polymerization of 300, with over 90% conversion observed for both monomers following 24 h (Fig. 2b and d). Previous work demonstrated the limitation of the synthetic pathway to neutrally charged RAFT monomers, with charged monomers either achieving either low conversion in the RAFT polymerization step, or causing the degradation of the PBAE, thus leading to the selection of NAM and DMA monomers for the following study.^25^ The resulting NAM_150_-(HDD-PIP)-NAM_150_ and DMA_150_-(HDD-PIP)-DMA_150_ polymers displayed *M*_n,SEC_ masses of around 39 000 g mol^−1^ (Fig. S1†). The dispersity values observed for both the polymer types prepared were higher than conventionally observed for RAFT polymers, with values of around 1.9 reported (Fig. 2d).^33^ This was hypothesised to originate from the broad molar mass distribution of the starting HDD-PIP polymer (dispersity of 1.43), synthesised through a step-growth polymerization step.

### QSI encapsulation and release

**Fig. 3:**
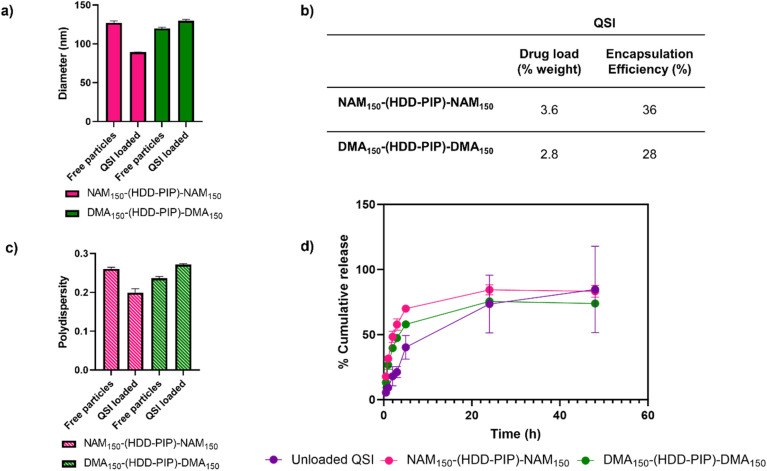
QSI encapsulation and release from NAM_150_-(HDD-PIP)-NAM_150_ and DMA_150_-(HDD-PIP)-DMA_150_ particles. (a) Change in mean particle diameter (nm) following QSI encapsulation, in water at 1 mg mL^−1^ where average diameter for QSI loaded particles is 89 nm for NAM_150_-(HDD-PIP)-NAM_150_ and 130 nm for DMA_150_-(HDD-PIP)-DMA_150_; (b) inset table that describes cumulative drug load (%) and encapsulation efficiency of QSI, defined by HPLC; (c) change in particle PdI following QSI encapsulation, in water at 1 mg mL^−1^ where average PdI for QSI loaded particles is 0.2 for NAM_150_-(HDD-PIP)-NAM_150_ and 0.27 DMA_150_-(HDD-PIP)-DMA_150_; (d) QSI release from NAM_150_-(HDD-PIP)-NAM_150_ (pink) and DMA_150_-(HDD-PIP)-DMA_150_ (green) particles and unloaded QSI (purple) in phosphate buffer pH 7.4, containing 0.01% Tween 20, at 37 °C, across 48 h, samples analysed by HPLC.

QSI encapsulation was achieved through simultaneous addition of water and a drug solution in DMSO to the required amount of dry polymer, followed by stirring for 2 h and subsequent removal of unencapsulated QSI by centrifugal filtration. A mean drug load of 3.6 ± 0.2% and 2.8 ± 0.2% was recorded for the NAM_150_-(HDD-PIP)-NAM_150_ and DMA_150_-(HDD-PIP)-DMA_150_ polymers respectively, across five experimental replicates (Fig. 3). QSI encapsulation yielded a slight change in the mean particle diameter with a decrease in particle diameter from 127 nm to 89 nm reported for NAM_150_-(HDD-PIP)-NAM_150_ particles and an increase from 120 nm to 130 nm reported for DMA_150_-(HDD-PIP)-DMA_150_. Particle polydispersity (PDI) remained within the 0.2–0.3 region with a decrease of PDI from 0.26 to 0.20 recorded for NAM_150_-(HDD-PIP)-NAM_150_ and an increase from 0.24 to 0.27 for DMA_150_-(HDD-PIP)-DMA_150_ particles. Considering previous reports of favourable biofilm penetration of particles with sizes within the 100–130 nm range,^34^ the diameters obtained for QSI loaded NAM_150_-(HDD-PIP)-NAM_150_ and DMA_150_-(HDD-PIP)-DMA_150_ particles were deemed as favourable for biofilm drug delivery. The decrease in NAM_150_-(HDD-PIP)-NAM_150_ particle diameter following QSI encapsulation was hypothesised to originate from the hydrophobic drug modifying the inner structure of the carrier, thus leading to changes in particle swelling. Previous work on the encapsulation of hydrophobic drugs in polyester particles demonstrated diameter changes were depended on the polymer's chemical composition and hydrophobicity, with particles composed of more hydrophobic polymers decreasing in diameter following drug encapsulation, while particles based on more hydrophilic materials swelled.^35^ Considering the NAM monomer is more hydrophobic (ChemDraw Clog *P* of −0.07) than DMA (ChemDraw Clog *P* of −0.17), these findings could explain the differences in diameter changes observed following QSI61 encapsulation in the polymeric carriers.

The *in vitro* release of the QSI from NAM_150_-(HDD-PIP)-NAM_150_ and DMA_150_-(HDD-PIP)-DMA_150_ micelles was evaluated in phosphate buffer at pH 7.4 (blood pH) simulating body temperature (37 °C) (Fig. 3). We observed QSI release from NAM_150_-(HDD-PIP)-NAM_150_ particles progressed at a faster rate with a cumulative drug release of 48% observed after 2 h and reaching 70% following 5 h. Comparatively the release of the QSI from DMA_150_-(HDD-PIP)-DMA_150_ particles progressed at a slightly slower rate achieving respectively 40 and 58% following 2 and 5 h. Cumulative release observed for the free drug control reached only 18% following 2 h, increasing to 40% after 5 h. Following 24 h a comparable cumulative release of around 75% was observed for the DMA_150_-(HDD-PIP)-DMA_150_ particles and the free drug control, while the amount of QSI released from NAM_150_-(HDD-PIP)-NAM_150_ particles reached 84%. The initial faster release of QSI from polymeric particles, compared to the free drug control may be attributed to association of the drug in micellar-like assemblies of the amphiphilic polymers released from regions of the kinetically-associated nanoparticles and passage of these across the membrane followed by dissociation on dilution in the receiver phase of the dialysis experiment. However, irrespective of the mechanism of transport, the overall effect was a burst release profile from the polymeric micelles, commonly observed for many nanoparticulate drug carriers.^36^ For some delivery systems, burst release is considered as undesirable as it can lead to systemic toxicity, for example when administering drugs such as anaesthetics or cytotoxic agents.^37,38^ However, for our target application the presence of a burst release profile was deemed desirable as it would lead to a rapid increase in QSI concentration at the biofilm site, hence preventing further biofilm maturation and rapidly potentiating the effect of the antibiotic. The presence of burst release further demonstrated the mechanism of QSI loading was based on its absorption onto the large surface of the particle rather than its incorporation within the particle core, which would result in a more sustained release profile.^39,40^ QSI release was shown to be independent of esterase action, achieving comparable cumulative release at equivalent timepoints to particles with no esterase presence (Fig. S3†). Considering the negligible esterase effect on cumulative drug release, we further hypothesised drug release occurred though diffusion from the polymeric particles, rather than through the erosion of the drug carrier.^41^

### Assessment of QS inhibition and antimicrobial activity

The ability of the encapsulated QSI to interfere with the *pqs* system was assessed through a reporter assay utilising a *P. aeruginosa* strain modified with a P_*pqsA*_-*lux* transcriptional fusion of the PqsA promoter (P_*pqsA*_) to the *luxCDABE* bioluminescence driving genes. The *P. aeruginosa pqs* system incorporates the LysR-type transcriptional regulator PqsR to control the expression of the *pqs* operon, the PQS-dependent activation of which is reported by P_*pqsA*_-*lux*. The pharmacological inhibition of PqsR by the QSI prevents the formation of the PqsR-PQS complex, therefore leading to a decrease in *lux* operon expression *via* detection of lower luminescence values. The QSI loaded NAM_150_-(HDD-PIP)-NAM_150_ and DMA_150_-(HDD-PIP)-DMA_150_ particles were tested at concentrations equivalent to 0.5, 5 and 50 μM encapsulated QSI against a positive control of free drug at corresponding concentrations, unloaded particle counterparts and a negative control of solvent vehicle (0.1% and 0.01% DMSO).

**Fig. 4:**
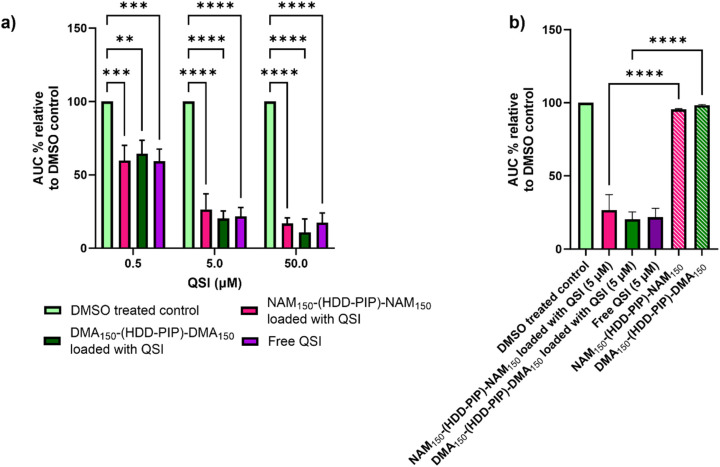
Assessment of QS inhibition in planktonic *P. aeruginosa*. (a) Activity comparison of particle loaded QSI and free QSI at concentration of 50, 5 and 0.5 μM to a DMSO control (0.1% and 0.01%) in bioreporter assay using PAO1-L mCTX::P_*pqsA*_-*lux*; (b) activity comparison of particle encapsulated QSI at 0.5 μM to not loaded NAM_150_-(HDD-PIP)-NAM_150_ and DMA_150_-(HDD-PIP)-DMA_150_ particles at equivalent concentrations in bioreporter assay using PAO1-L mCTX::P_*pqsA*_-*lux*. All measurements were performed in triplicate apart from non-loaded particles which were tested in duplicate, using biologically independent replicates and the error bars represent the mean ± standard deviation. Statistical testing was performed with a one-way ANOVA followed by a *post-hoc* Tukey test to identify individual comparisons. Statistical significance is represented as *p < 0.05, **p < 0.01, ***p < 0.001, ****p < 0.0001.

Successful release of the encapsulated QSI from NAM_150_-(HDD-PIP)-NAM_150_ and DMA_150_-(HDD-PIP)-DMA_150_ particles was demonstrated with comparable inhibition of P_*pqsA*_-*lux* transcriptional fusion observed between the free and encapsulated QSI at all the concentrations tested (Fig. 4a and S4†). This corresponded to a reduction of the activation of the P_*pqsA*_-*lux* transcriptional fusion by over 80%, compared to untreated control, when administered at concentrations corresponding to 50 μM, and a reduction of over 70% and over 30% at 5 and 0.5 μM respectively. Considering particle encapsulation was not expected to enhance QSI efficacy, the results obtained fit within our initial predictions. RAFT-PBAE-RAFT particles not loaded with QSI showed no inhibitory effect against P_*pqsA*_-*lux*, confirming the *pqs* inhibition originated from QSI release (Fig. 4b).

The antimicrobial efficacy of NAM_150_-(HDD-PIP)-NAM_150_ and DMA_150_-(HDD-PIP)-DMA_150_ polymeric particles was evaluated in a checkerboard assay with and without the presence of QSI (concentrations ranging from 10 to 1.25 μM), in planktonic *P. aeruginosa* cultures, starting at polymer concentrations of 1 mg mL^−1^. Over 60% bacterial survival was observed for all the polymer/QSI concentrations tested, with no potentiation of the polymers antimicrobial action observed following QSI addition (Fig. S5†). Hence the antimicrobial effect of both the polymers was deemed as negligible, with the observed differences in luminescence attributed solely to QSI activity.

### Assessment of antibiofilm activity of the QSI loaded particles

**Fig. 5:**
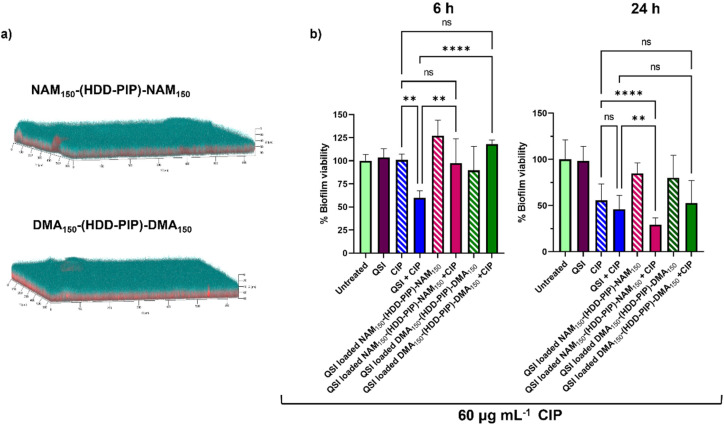
Biofilm penetration and QSI activity in *P. aeruginosa* biofilms. (a) Representative confocal laser scanning microscopy (CLSM) images the biofilm penetration of rhodamine-tagged NAM_150_-(HDD-PIP)-NAM_150_ and DMA_150_-(HDD-PIP)-DMA_150_ particles (red) in mature *P. aeruginosa* PA01L biofilms (1-day old) stained with SYTO9 (blue) following a 4 h incubation, additional images are provided in Fig. S7;† (b) bar charts showing viability in PAO1-L biofilms quantified after treatment with different conditions for 6 and the 24 h. The concentrations of the drugs used were CIP- 60 μg mL^−1^ and free QSI or particle loaded QSI at 10 μM. All measurements were performed in triplicate, using biologically independent replicates and the error bars represent the mean ± standard deviation. Statistical testing was performed with a one-way ANOVA followed by a *post-hoc* Tukey test to identify individual comparisons. Statistical significance is represented as *p < 0.05, **p < 0.01, ***p < 0.001, ****p < 0.0001.

The primary aim of QSI encapsulation within polymeric micelles was the improvement of the QSIs biofilm penetration and therefore efficacy. To evaluate the potential of NAM_150_-(HDD-PIP)-NAM_150_ and DMA_150_-(HDD-PIP)-DMA_150_ particles for biofilm drug delivery we first tested the diffusion of the nanoparticles through the biofilm matrix in mature 1-day old *P. aeruginosa* biofilms (∼70 μm depth). Biofilms were exposed to Rhodamine B labelled NAM_150_-(HDD-PIP)-NAM_150_ and DMA_150_-(HDD-PIP)-DMA_150_ analogues and the fluorescence signal measured at different layers of the biofilm depth using confocal microscopy (Fig. 5a). Following a 4 h incubation time we observed biofilm saturation with the tagged particles particularly at levels below 50 μm, confirming particle suitability for QSI delivery to bacterial biofilms.

We then evaluated the antimicrobial potentiating effect that QSI loaded particles exhibited on mature *P. aeruginosa* biofilms in combination with CIP (60 μg mL^−1^). Considering our previous work on polymer–QSI conjugates, where a significant efficacy improvement over free drug was observed following 6 h of exposure, we evaluated biofilm survival at 6 h and 24 h following treatment.^23^

Interestingly we observed a negligible reduction in biofilm viability following 6 h, for both NAM_150_-(HDD-PIP)-NAM_150_ and DMA_150_-(HDD-PIP)-DMA_150_ encapsulated QSI particles when co-administered with 60 μg mL^−1^ CIP (×300 the MIC of planktonic *P. aeruginosa* cells^42^), and with less than a 10% reduction in biofilm viability observed for both particle variants, compared to an average 40% viability reduction for the non-encapsulated QSI administered with CIP (Fig. 5b). Following 24 h the effect of particle encapsulation on QSI efficacy was shown to increase, particularly for QSI encapsulated in NAM_150_-(HDD-PIP)-NAM_150_, reaching an over 70% reduction in biofilm viability when administered with CIP. This achieved a statistically significant improvement (adjusted *p* value of 0.0077) upon a 54% reduction in biofilm viability observed for the free QSI and CIP combination treatment. QSI loaded DMA_150_-(HDD-PIP)-DMA_150_ particles were shown to potentiate the effect of CIP to a lesser extent, reaching an average 47% reduction in biofilm viability following 24 h, hence not improving upon free QSI efficacy. For CIP administered without the addition of QSI an average 44% reduction in biofilm viability was observed, demonstrating QSI presence does potentiate antibiotic effect. This was particularly significant for QSI encapsulated in NAM_150_-(HDD-PIP)-NAM_150_ particles with an adjusted p value below 0.0001. To further confirm the potentiation of CIP antibiofilm efficacy originated from the inhibition of QS, rather than the synergy of the antibiotic with QSI61, a checkerboard assay of the two compounds was conducted in planktonic *P. aeruginosa*, with no synergy observed (Fig. S6†).

We hypothesise the higher efficacy observed for QSI encapsulated in NAM_150_-(HDD-PIP)-NAM_150_ particles originated from their faster release profile, reaching 70% cumulative release following 5 h, compared to 58% reported for DMA_150_-(HDD-PIP)-DMA_150_ particles. Considering the QSI must be released from the polymeric particles in order to potentiate antibiotic activity, a faster release profile provides a higher quantity of QSI in a shorter time period, hence enhancing the therapeutic effect to a higher extent. This further explains the lack of efficacy observed for the particle loaded QSI following 6 h, with insufficient time available for the release and subsequent activity of QSI61. No reduction in biofilm viability was observed for QSI-loaded NAM_150_-(HDD-PIP)-NAM_150_ and DMA_150_-(HDD-PIP)-DMA_150_ particles when CIP was not present (Fig. 5b) and for empty particles without QSI encapsulated (Fig. S8a†). A further evaluation of non-QSI loaded polymeric particles administered in combination with CIP (60 μg mL^−1^) (Fig. S8b†), demonstrated no significant improvement of antibiotic action when administered together with the polymer, hence showing the potentiation of CIP efficacy originated from the improvement of QSI61 biofilm penetration.

This confirms the antibiofilm activity observed resulted from the potentiation of antibiotic effect, rather than the antimicrobial nature of the polymeric particles or the QSI.

Our findings indicate biofilm susceptibility was influenced by the greater penetration of QSI when encapsulated in polymeric particles. However, the release rate of the encapsulated cargo was shown as imperative to QSI activity, with particles with a faster release profile achieving a higher potentiation of CIP efficacy. Moreover, the high resistance to CIP observed throughout the course of the study demonstrates an urgent need for effective drug delivery platforms, with our RAFT-PBAE-RAFT particles offering an effective approach towards the delivery of hydrophobic *pqs* antagonists.

## Conclusions

In conclusion, we demonstrate a new approach to aid QSI penetration through *P. aeruginosa* biofilms using RAFT-PBAE-RAFT particles. Following successful QSI encapsulation in NAM_150_-(HDD-PIP)-NAM_150_ and DMA_150_-(HDD-PIP)-DMA_150_ particles we showed effective drug release and inhibition of the bacterial *pqs* system using a luminescence bioreporter assay, with comparable *pqs* inhibition observed between the particle-encapsulated and non-encapsulated QSI. We then verified rhodamine-tagged polymeric particles can penetrate into deep regions of thick *P. aeruginosa* biofilms, hence enabling a significant increase in the potentiation of CIP (60 μg mL^−1^) activity. This was particularly visible for QSI loaded in NAM_150_-(HDD-PIP)-NAM_150_ particles, significantly improving upon the activity of the non-encapsulated QSI and CIP combination treatment. Our findings demonstrate a substantial effect of the QSIs release profile on its efficacy, with the faster releasing NAM_150_-(HDD-PIP)-NAM_150_ particles achieving a significantly higher potentiation of CIP effect, compared to DMA_150_-(HDD-PIP)-DMA_150_. Hence, further research into alternative RAFT-PBAE-RAFT structural conformations could provide additional improvements upon the reported results, provided faster release profiles are achieved.

In comparison to previously reported methods on QSI61 delivery, by encapsulating the QSI in the particles hydrophobic PBAE core, rather than covalently binding it to an amphiphilic polymer chain, we achieved an improved inhibition of the *lux* operon, by making QSI activity independent of esterase presence. This was particularly visible when applying the QSI at lower concentrations of 0.5 and 5 μM, with comparable *pqs* inhibition observed for both the encapsulated QSI and the native QSI molecular control.

Our findings demonstrate RAFT-PBAE-RAFT particles are an attractive approach to the delivery of hydrophobic QSI molecules, enabling an enhanced efficacy of the encapsulated drug. Considering the promising results observed within this study, further work into the encapsulation of the antibiotic used within the combination therapy could yield additional efficacy improvements, through a simultaneous enhancement of the antibiotics penetration through the bacterial biofilm.

## Data availability

All relevant data can be obtained upon request from the authors at cameron.alexander@nottingham.ac.uk.

## Conflicts of interest

We declare no conflicts of interest.

## Supplementary Material

LP-002-D3LP00208J-s001
