# Supplementary material for: Triblock copolymer micelles enhance solubility, permeability and activity of a quorum sensing inhibitor against Pseudomonas aeruginosa biofilms
Source: RSC Appl Polym. 2024 Feb 27;2(3):444–55. doi: 10.1039/d3lp00208j (PMC11114570; doi:10.1039/d3lp00208j)
Supplement: LP-002-D3LP00208J-s001 [file LP-002-D3LP00208J-s001.pdf]

Electronic Supplementary Material (ESI) for RSC Applied Polymers.
This journal is © The Royal Society of Chemistry 2024

Supplementary information

Triblock copolymer micelles enhance solubility, permeability and activity of a quorum sensing inhibitor against Pseudomonas aeruginosa biofilms

Karolina Kasza[a,b], Fadi Soukarieh[b], Manuel Romero[b,c], Kim R. Hardie[b], Pratik Gurnani[d], Miguel Cámara[b], and Cameron Alexander*[a]

[a] Division of Molecular Therapeutics and Formulation, School of Pharmacy, University of Nottingham, NG7 2RD, UK

[b] National Biofilms Innovation Centre, School of Life Sciences, Biodiscovery Institute, University Park, University of Nottingham, Nottingham NG7 2RD, UK

[c]Department of Microbiology and Parasitology, Faculty of Biology-CIBUS, Universidade de Santiago de Compostela, Santiago de Compostela, 15782, Spain.

[d]UCL School of Pharmacy, University College London, 29-39 Brunswick Square, London, WC1N 1AX, UK

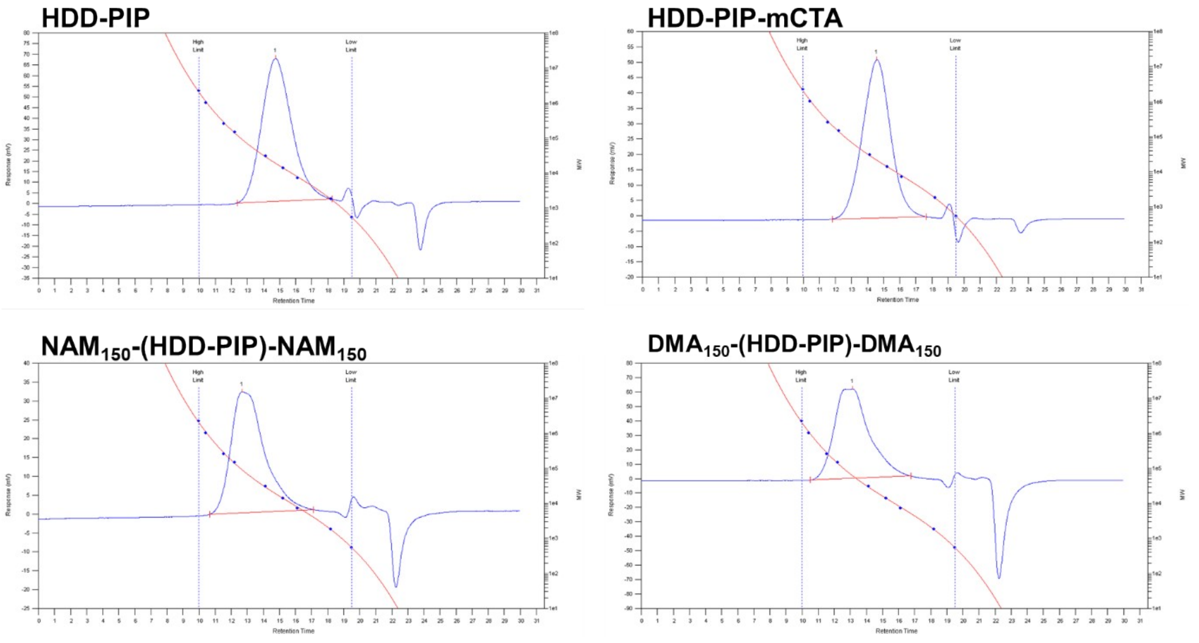

Figure S1: DMF-SEC chromatograms of prepared polymers.

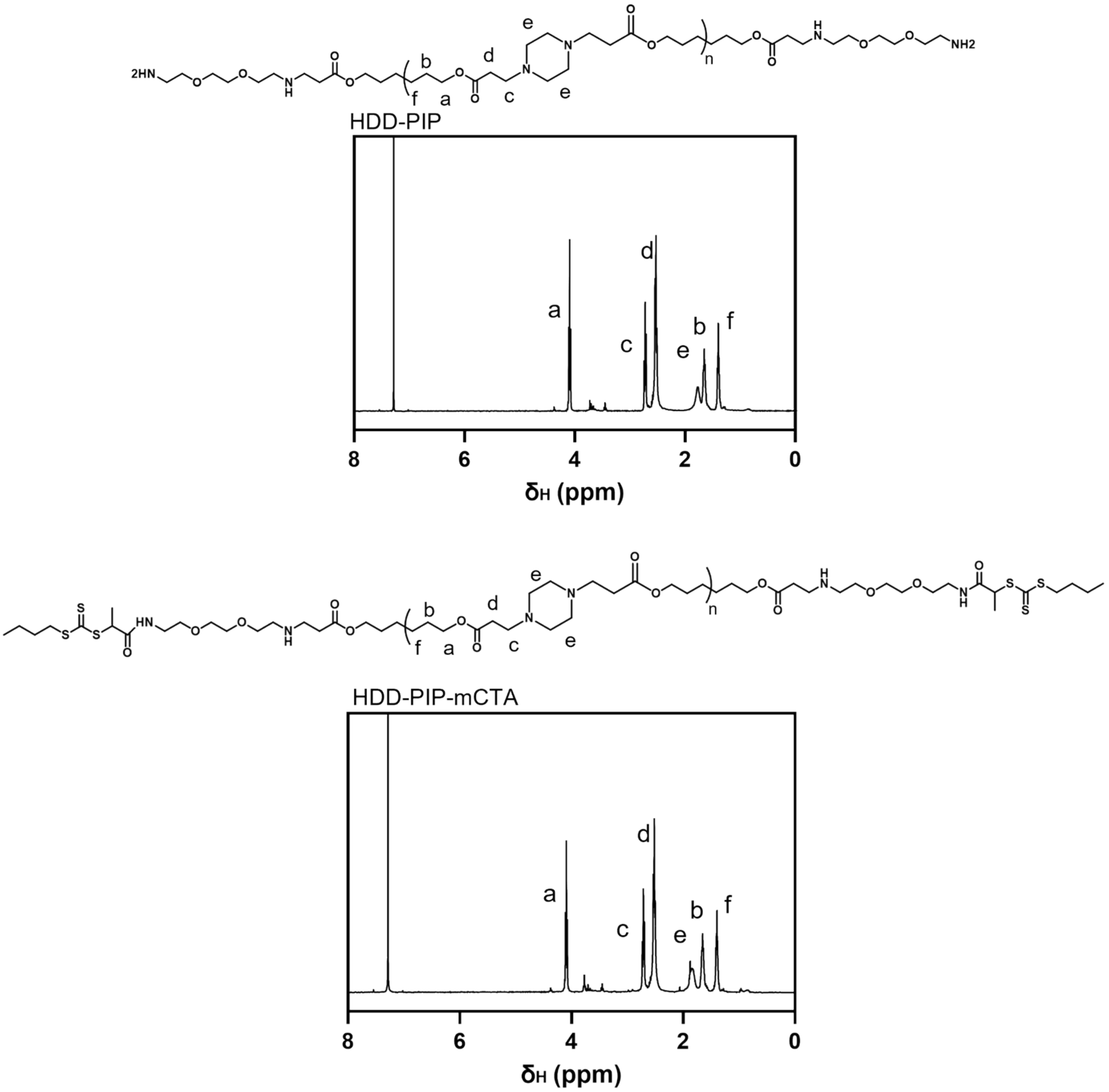

Figure S2: ^{1}H NMR spectra of polymers in chloroform-d recorded at 25°C with a Bruker NMR400 MHz.

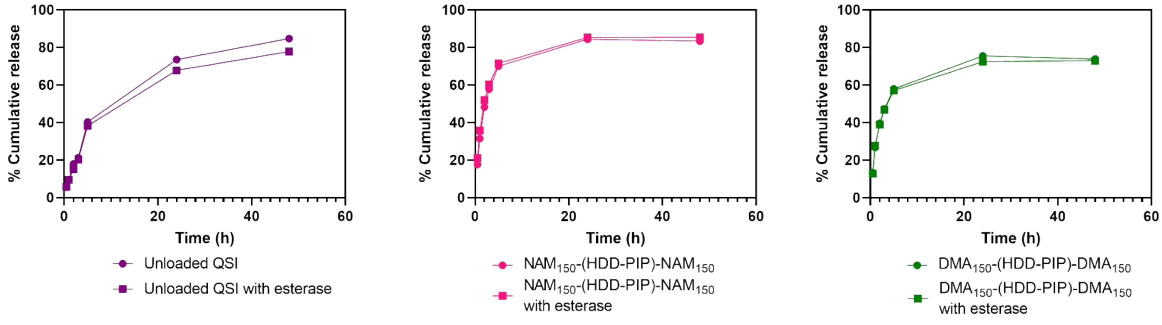

Figure S3: QSI release from NAM_{150}-(HDD-PIP)-NAM_{150} (pink) and DMA_{150}-(HDD-PIP)-DMA_{150} (green) particles and unloaded QSI (purple) in phosphate buffer pH 7.4, containing

0.01% Tween 20, at 37˚˚C, across 48 h, with and without esterase presence samples analysed by HPLC.

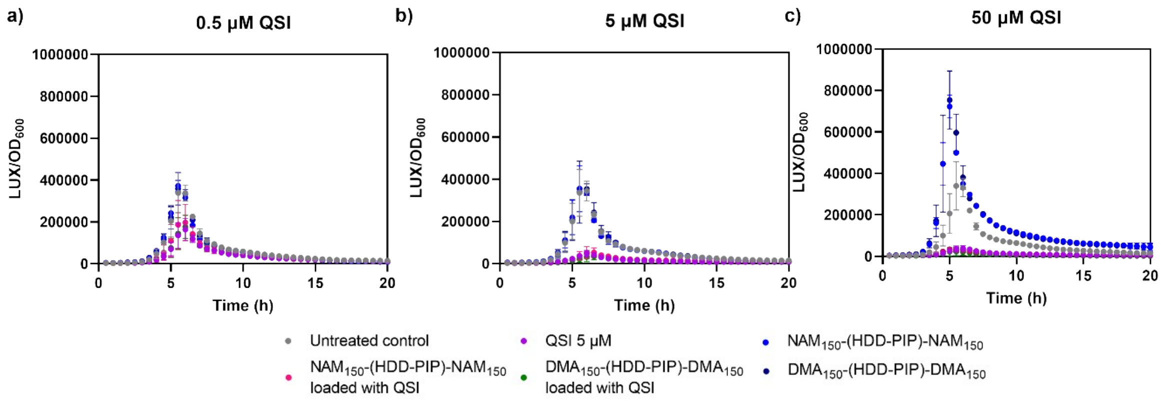

Figure S4: Effect of QSI and QSI loaded polymers on a P_{pqsA}-*lux* transcriptional fusion, which reports the PQS-dependent activation of the *pqs* operon mediated by PqsR. **a)** QSI concentrations at 0.5 µM QSI and non-loaded particles at corresponding concentrations; **b)** QSI concentrations at 5 µM QSI and non-loaded particles at corresponding concentrations; **c)** QSI concentrations at 50 µM QSI and non-loaded particles at corresponding concentrations. Values given are averages from three different cultures for QSI activity testing and two different cultures for the testing of non-loaded polymeric particles and correspond to the relative light units normalized to culture density (Lux/OD_{600}) over time (20 h).

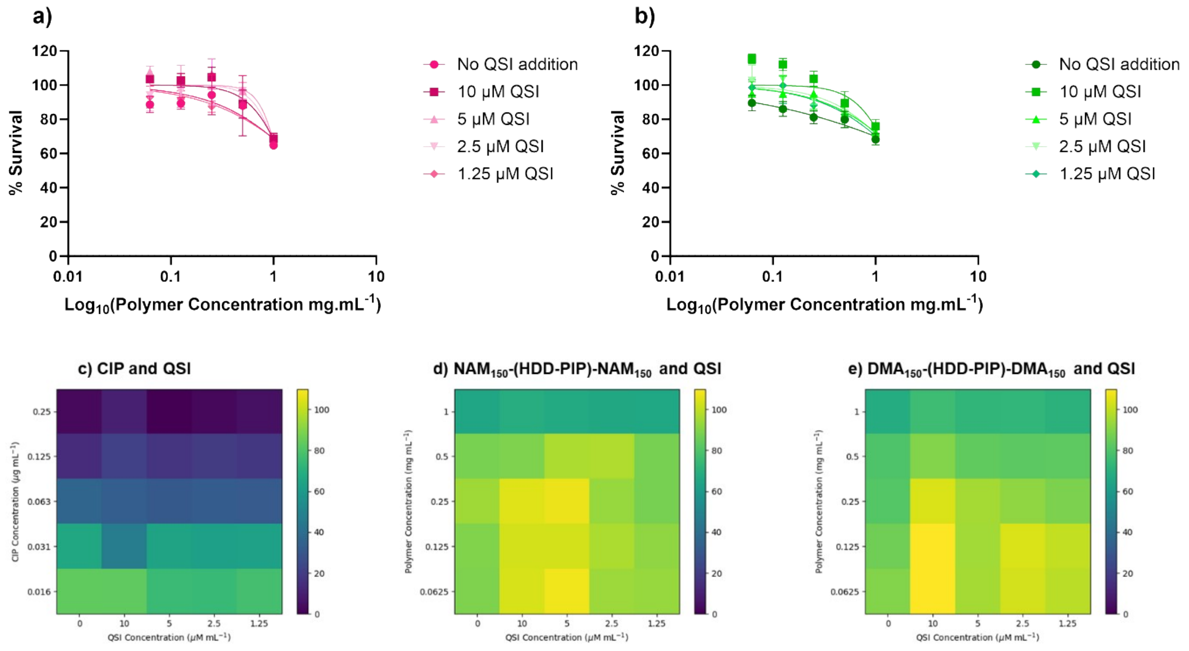

Figure S5: Antimicrobial activity of polymeric particles with and without QSI61 addition. **a)** Concentration curves of NAM_{150}-(HDD-PIP)-NAM_{150} (pink) with and without QSI61 addition against planktonic *P. aeruginosa*; **b)** Concentration-activity curves of DMA_{150}-(HDD-PIP)-DMA_{150} (green) with and without QSI61 addition against planktonic *P. aeruginosa*. Heat maps to show effects of combinations of **c)**, CIP +QSI, **d**, NAM_{150}-(HDD-PIP)-NAM_{150} and QSI, and **e**) DMA_{150}-(HDD-PIP)-DMA_{150} and QSI.

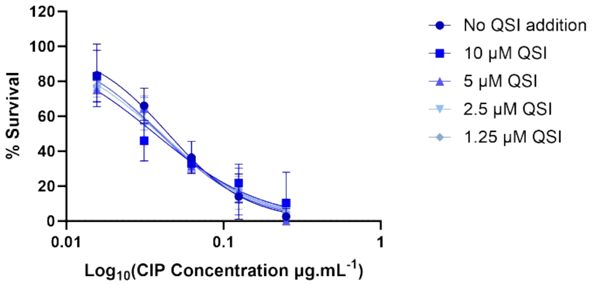

Figure S6: CIP concentration-activity curves with and without QSI61 addition against planktonic *P. aeruginosa*.

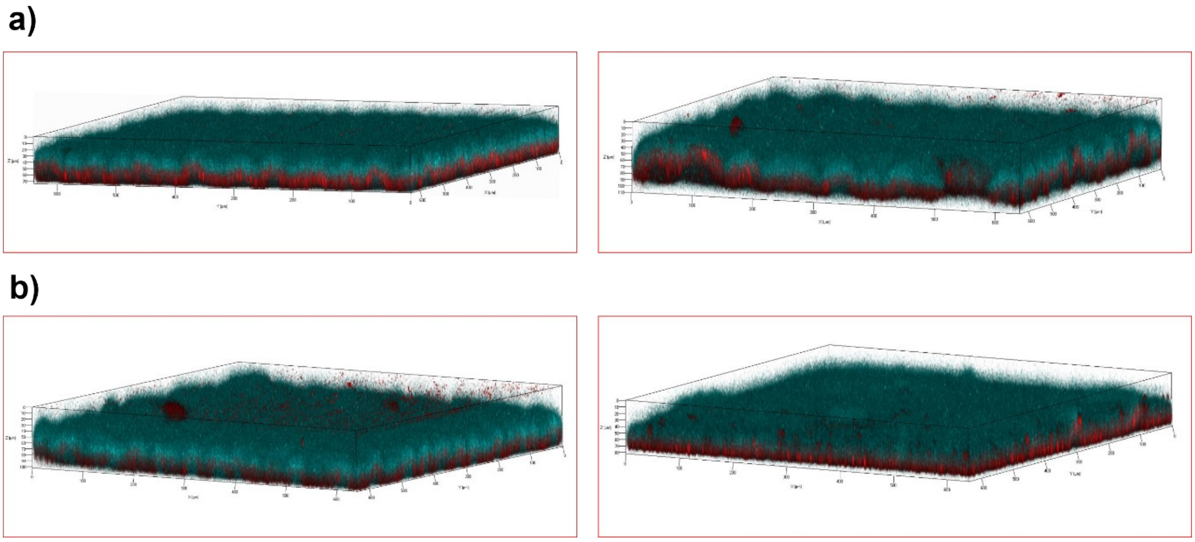

Figure S7: Confocal laser scanning microscopy (CLSM) images the biofilm penetration of rhodamine-tagged DMA_{150}-(HDD-PIP)-DMA_{150} (**a**) and NAM_{150}-(HDD-PIP)-NAM_{150} particles (**b**) (red) in mature *P. aeruginosa* PA01L biofilms (1-day old) stained with SYTO9 (blue) following a 4 h incubation.

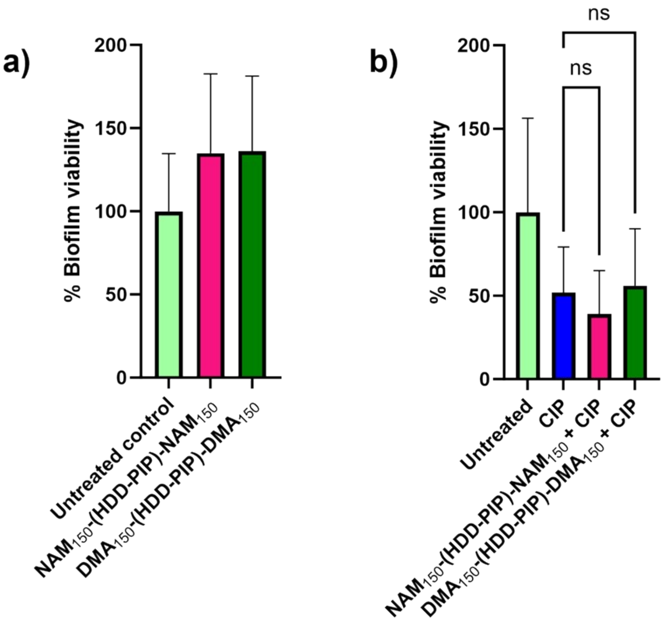

Figure S8: Antimicrobial activity of polymeric particles in *P. aeruginosa* biofilms; **a)** Bar charts showing viability in mature *P. aeruginosa* biofilms, quantified after treatment with NAM_{150}-(HDD-PIP)-NAM_{150} (pink) and DMA_{150}-(HDD-PIP)-DMA_{150} (green) particles with no encapsulated QSI; measurements were performed in duplicate, using biologically independent replicates and the error bars represent the mean ± standard deviation; **b)** Bar charts showing viability in mature *P. aeruginosa* biofilms, quantified after treatment with CIP (60 μg mL^{-1}) and NAM_{150}-(HDD-PIP)-NAM_{150} (pink) and DMA_{150}-(HDD-PIP)-DMA_{150} (green) particles with no encapsulated QSI administered in combination with CIP (60 μg mL^{-1}); measurements are an average of 15 coverslip measurements using one biological replicate and the error bars represent the mean ± standard deviation. Statistical testing was performed with a one-way ANOVA followed by a post-hoc Tukey test to identify individual comparisons. Statistical significance is represented as *$p < 0.05$, **$p <0.01$, ***$p < 0.001$, ****$p < 0.0001$.
